# Derivation and validation of a prognostic model for postoperative risk stratification of critically ill patients with faecal peritonitis

**DOI:** 10.1186/s13613-017-0314-1

**Published:** 2017-09-12

**Authors:** Ascanio Tridente, Julian Bion, Gary H. Mills, Anthony C. Gordon, Geraldine. M. Clarke, Andrew Walden, Paula Hutton, Paul A. H. Holloway, Jean-Daniel Chiche, Frank Stuber, Christopher Garrard, Charles Hinds, On behalf of the GenOSept and GAinS Investigators

**Affiliations:** 1 Whiston Hospital Prescot, Merseyside and Department of Infection, Immunity and Cardiovascular Disease, The Medical School, University of Sheffield, Sheffield, UK; 2 School of Clinical and Experimental Medicine, University of Birmingham, Birmingham, UK; 3 University of Sheffield, Sheffield, UK; 4 Imperial College, London, UK; 5 The Wellcome Trust Centre for Human Genetics, University of Oxford, Oxford, UK; 6 Intensive Care Unit, Royal Berkshire Hospital, Reading, UK; 7 Intensive Care Unit, John Radcliffe Hospital, Oxford, UK; 8 Hospital Cochin, Paris, France; 9 Department of Anaesthesiology and Pain Medicine, Bern University Hospital and University of Bern, Bern, Switzerland; 10 Barts and the London Queen Mary School of Medicine, London, UK

**Keywords:** Faecal peritonitis, Outcome, Prognostication, GenOSept, GAinS, Sepsis

## Abstract

**Background:**

Prognostic scores and models of illness severity are useful both clinically and for research. The aim of this study was to develop two prognostic models for the prediction of long-term (6 months) and 28-day mortality of postoperative critically ill patients with faecal peritonitis (FP).

**Methods:**

Patients admitted to intensive care units with faecal peritonitis and recruited to the European GenOSept study were divided into a derivation and a geographical validation subset; patients subsequently recruited to the UK GAinS study were used for temporal validation. Using all 50 clinical and laboratory variables available on day 1 of critical care admission, Cox proportional hazards regression was fitted to select variables for inclusion in two prognostic models, using stepwise selection and nonparametric bootstrapping sampling techniques. Using Area under the receiver operating characteristic curve (AuROC) analysis, the performance of the models was compared to SOFA and APACHE II.

**Results:**

Five variables (age, SOFA score, lowest temperature, highest heart rate, haematocrit) were entered into the prognostic models. The discriminatory performance of the 6-month prognostic model yielded an AuROC 0.81 (95% CI 0.76–0.86), 0.73 (95% CI 0.69–0.78) and 0.76 (95% CI 0.69–0.83) for the derivation, geographic and temporal external validation cohorts, respectively. The 28-day prognostic tool yielded an AuROC 0.82 (95% CI 0.77–0.88), 0.75 (95% CI 0.69–0.80) and 0.79 (95% CI 0.71–0.87) for the same cohorts. These AuROCs appeared consistently superior to those obtained with the SOFA and APACHE II scores alone.

**Conclusions:**

The two prognostic models developed for 6-month and 28-day mortality prediction in critically ill septic patients with FP, in the postoperative phase, enhanced the day one SOFA score’s predictive utility by adding a few key variables: age, lowest recorded temperature, highest recorded heart rate and haematocrit. External validation of their predictive capability in larger cohorts is needed, before introduction of the proposed scores into clinical practice to inform decision making and the design of clinical trials.

**Electronic supplementary material:**

The online version of this article (doi:10.1186/s13613-017-0314-1) contains supplementary material, which is available to authorized users.

## Background

Prognostic scores and models of illness severity are useful both clinically and for research. They support critical care physicians in decision making through more accurate prognostication; they describe and summarise case mix, and inform health economic evaluations of cost-effectiveness. Many types of models exist, and their roles are not mutually exclusive, as their combined use may afford better prognostic reliability [1]. These tools are usually insufficiently accurate to be useful for predicting individual survival and are generally reserved for benchmarking quality of care and for research studies [2–4], for example when examining heterogeneity of treatment effect in clinical trials [5].

When considering prognostication in the context of the wide ranging spectrum of intra-abdominal infections, complexity is increased by the heterogeneity of aetiology, clinical manifestations and pathophysiological mechanisms. The International Sepsis Forum Consensus Conference on Definitions of Infection in the Intensive Care Unit describes intra-abdominal infections as a “very heterogeneous group of infectious processes that share an anatomical site between the diaphragm and the pelvis” [6]. The anatomical, clinical and pathophysiological heterogeneity of these infections, together with their varied aetiology and prognosis, have given rise to a range of prognostic instruments tailored to specific populations.

Generic “peritonitis” prognostic tools (aimed at peritonitis of any origin), such as the Mannheim Peritonitis Index (MPI) or the Peritonitis Index of Altona II (PIA II), rely on factors such as age, degree of organ failure, origin of sepsis and intra-operative findings to risk-stratify different types of peritonitis, but, given the considerable heterogeneity of intra-abdominal infections, these scoring systems may not be sufficiently specific in terms of aetiology [7, 8]. Other scoring systems have been devised to explicitly address the issue of prognostication in selected forms of peritonitis, such as the left colonic Peritonitis Severity Score (PSS), developed for patients with distal large bowel peritonitis of various origins [9]. The physiological and operative severity score for the enumeration of mortality and morbidity (POSSUM) is another risk adjustment model, developed in 1991 for use in surgical patients [10]. A modification of this prognostic model, obtained by excluding some of the physiological factors of the original POSSUM, was developed for use specifically in patients undergoing surgery for colorectal cancer (CR-POSSUM) [11]. Importantly, all of these scores incorporate intra-operative findings and are either designed to cater for, and include, the whole heterogeneous spectrum of peritoneal infections (such as the MPI and PIA II), or to focus on a very narrow subset of peritonitis, identified by location (left colonic, in the case of PSS) or aetiology (colorectal malignancy, as in CR-POSSUM).

To date no prognostic score has been developed for the critically ill patient with faecal peritonitis (FP) in the postoperative phase. We therefore aimed to specifically study critically ill patients suffering from FP, in the postoperative phase, and quantify their mortality risk at 28 days and 6 months. International multicentre prospectively collected patient datasets, such as The GenOSept and GAinS cohorts, provided an opportunity to develop and evaluate such prognostic systems.

## Methods

### Aim, design and setting

The Genetics of Sepsis and Septic Shock in Europe (GenOSept) and Genomic Advances in Sepsis (GAinS) are prospectively gathered cohorts of critically ill septic patients with FP recruited from multiple centres in Europe. They include data from patients with various degrees of illness severity, including potential risk modifiers and confounding factors (such as comorbidities, indices of acute physiological derangement, organ support, radiological and laboratory findings, origin of FP) [12, 13]. These diagnostically homogeneous cohorts of FP patients, gathered primarily for the purposes of studying genetic epidemiology in sepsis, also provide high-quality data well suited to the development and testing of a prognostic model specific to this postoperative patient population.

The primary aim of this study was to develop and validate a prognostic modelling tool able to stratify postsurgical critically ill patients with FP, by quantifying their mortality risk in the short- (28 day) and long-term (6 month), independently from intra-operative surgical findings, using prospectively collected data from the GenOSept and GAinS cohorts.

### Recruitment criteria

The same inclusion and exclusion criteria were used for both cohorts. Inclusion criteria: adult patients (>18 years) admitted to a High Dependency Unit (HDU) or Intensive Care Unit (ICU) with FP, defined as visible inflammation of the serosal membrane that lines the abdominal cavity, secondary to contamination by faeces, as diagnosed by the operating surgeon at laparotomy. All critically ill patients in this cohort, therefore, were recruited after the diagnosis was established during surgical source control. Exclusion criteria: peritonitis due to gastric or upper GI-tract perforation (e.g. gastric or duodenal ulcer perforation, small bowel perforation), patient or legal representative unwilling or unable to give consent; patient pregnant; advanced directive to withhold or withdraw life-sustaining treatment or admitted for palliative care only; patient already enrolled in an interventional research study of a novel/unlicensed therapy (patients enrolled in interventional studies examining the clinical application or therapeutic effects of widely accepted, “standard” treatments, were not excluded); patient immunocompromised (known regular systemic corticosteroid therapy, exceeding 7 mg/kg/day of hydrocortisone or equivalent, within 3 months of admission and prior to acute episode; known regular therapy with other immunosuppressive agents, e.g. azathioprine; known to be HIV positive or have acquired immunodeficiency syndrome as defined by the Centre for Disease Control; neutrophil count less than 1000 mm^−3^ due to any cause, including metastatic disease and haematological malignancies or chemotherapy, but excluding severe sepsis; organ or bone marrow transplant receiving immunosuppressive therapy).

The definition of sepsis was based on the International Consensus Criteria: “the clinical syndrome defined by the presence of both infection and a systemic inflammatory response” [14]. Patients were followed for up to 6 months from enrolment or until death.

### Database and quality assurance

The case report form (CRF) was developed and tested by CH, CG, AG, JDC and Dr J. Millo, together with other members of the GenOSept Consortium. Variables recorded included demographic, clinical and outcome data. A specific electronic case report form (eCRF) was developed by Lincoln, Paris, France, using software developed in collaboration with JDC. The database was password-protected, allowing investigators to enter data into the eCRF online, and included audit trail capability for data entry and subsequent modifications. To minimise errors, logical range checks were in place so that the investigators would be alerted if an attempt was made to enter data values outside the expected ranges.

Quality assurance (QA) was performed by P.H., C.G., A.W., A.G. and C.H, who systematically reviewed all data. Data queries (DQs) were generated within the eCRF for missing or erroneous data and sent electronically to the relevant investigators for action, where necessary. Up to the end of January 2011, an estimated 3986 valid DQs had been generated, with a response rate by the investigators of approximately 92%. Common reasons for DQs were missing information, particularly the Charlson Index, antimicrobial use, estimated day of onset of FP before ICU admission, information about circumstances of GCS assessment and outcome data.

All patients’ eCRFs were reviewed by experienced critical care physicians. Where the patient’s eligibility for inclusion in the relevant cohort was unclear, clarification was sought from the investigators. Regular QA reports were provided to the relevant Management Committee for review; the National Investigators were contacted regarding quality issues if necessary.

### Statistical analyses

#### Prognostic model

In order to build the prognostic model, patients recruited up to January 2011 (included in the GenOSept cohort) were divided into two subsets of patients: one for derivation and the other for external geographic validation. To limit the effect of potentially unmeasured and unaccounted confounding factors, related to possible differences in national systems of healthcare provision among participating countries across Europe, these patients were divided into UK (derivation) and non-UK (geographic validation) sub-cohorts, with the aim of optimising homogeneity in the datasets and decreasing potential background noise. Subsequent patients recruited in the UK between January 2011 and March 2015 (included in the GAinS cohort) were included in the temporal validation cohort.

We evaluated all 50 clinical and laboratory variables available on admission to critical care (day 1) (for a full list, see Additional file 1). The primary outcome was 6-month mortality risk with the secondary outcome being 28-day mortality risk. To select the variables to include in the model, Cox proportional hazards regression analysis for 6-month mortality was fitted, using stepwise backwards selection, to determine the predictors to be included in the models from 50 bootstrapped samples derived from the derivation subset (nonparametric bootstrap procedure). Increasing the number of bootstrap replications did not alter the model significantly. The p value cut-off used was 0.05. The same predictor variables were employed to construct a prognostic tool for the secondary outcome, 28-day mortality.

The procedure of bootstrapping is a re-sampling method which relies on random sampling with replacement of the available observations. This procedure allows evaluation of the characteristics of an estimator (such as its variance) by measuring those properties when obtaining multiple samples from the original dataset (and of size equal to the observed dataset) [15, 16].

A final Cox proportional hazards regression analysis for both 6-month and 28-day mortalities was fitted using the set of variables found to be significant in the majority of bootstrap replications.

We confirmed that the proportional hazards assumption was met by drawing Kaplan–Meier Curves and Nelson Aalen plots for the covariates after categorisation. Predictors which satisfy the proportional hazard assumption show very similar curves, with the separation between them remaining proportional across analysis time [17]. We also tested the correctness of this assumption testing on the basis of Schoenfeld residuals [18].

In order to assess for the presence of collinearity (which happens when two variables are almost perfect linear combinations of one another), we calculated the variance inflation factors (VIFs). It is generally accepted that variables with VIFs greater than 10 merit further investigation [19].

The two models obtained were evaluated using area under the receiver operating characteristic curve (AuROC) analysis, which plots sensitivity against 1-specificity to describe the accuracy of a diagnostic test [20, 21] and to compare the performance of different tests [22].

#### Nonparametric bootstrapping and prognostic model derivation for 6-month mortality

The bootstrapping procedure was performed using 50 repetitions based on the UK derivation cohort. A final Cox proportional hazards regression analysis for 6-month mortality was fitted using the set of variables found to be significant in the majority of bootstrap replications. Saturation was reached after 50 bootstrap replications, with additional replications not yielding significantly different results.

A set of 5 variables assessed on day 1 met this criterion (age, SOFA score, lowest temperature, highest heart rate, haematocrit). The Cox proportional hazards model estimates for those risk variables are presented in Table 1.

**Table 1:** Variables found to be significant in the majority of bootstrap replications run on the UK derivation cohort for the two outcomes

Variable	HR	HR 95% CI	coeff	coeff 95% CI	*p*
6 month mortality					
Age	1.05	1.03–1.07	0.045	0.02–0.06	<0.001
SOFA score	1.20	1.12–1.28	0.18	0.11–0.25	<0.001
Low temperature	0.76	0.63–0.91	[−0.28]	[−0.46]–[−0.09]	0.004
High heart rate	1.01	1.01–1.02	0.01	0.01–0.02	0.007
Haematocrit	0.97	0.94–0.99	[−0.031]	[−0.059]–[−0.003]	0.028
28-day mortality					
Age	1.05	1.03–1.08	0.049	0.03–0.07	<0.001
SOFA score	1.22	1.12–1.33	0.2	0.11–0.29	<0.001
Low temperature	0.70	0.55–0.88	[−0.36]	[−0.59]–[−0.13]	0.002
High heart rate	1.01	1–1.02	0.01	[−0.001]–0.2	0.07
Haematocrit	0.99	0.95–1.02	[−0.013]	[−0.047]–0.022	0.47

*HR* hazard ratio; *95% CI* 95% confidence interval, *coeff* coefficient, *SOFA* Sequential Organ Failure Assessment; the use of the square brackets [] indicates negative values

The same five variables were employed to formulate the 6-month mortality prognostic tool by entering the estimates obtained from the Cox proportional hazards model in the following equation:

$${\text{FP}}\,{\text{score}}\,\left( {6\,{\text{month}}} \right) = \left( {10^{3} } \right) * \exp \left( {\left( {0.0447387*A} \right) + \left( {0.1812872*S} \right) + \left( { - 0.2767377*T} \right) + \left( {0.0114629*{\text{HR}}} \right) + \left( { - 0.0313029*H} \right)} \right)$$

where *A* = age at admission to critical care, *S* = SOFA score day 1, *T* = lowest recorded temperature (as °C) on day 1, HR = highest recorded heart rate on day 1, *H* = haematocrit (as percentage points) on day 1.

The model coefficients used for prediction of 6-month mortality were adjusted for the 28-day mortality outcome. To achieve this, a separate Cox proportional hazards regression analysis was fitted for 28-day mortality, utilising the same set of five variables. The resulting model estimates are presented in Table 1. The estimates were utilised to construct the 28-day mortality prognostic tool as described in the following equation:

$${\text{FP}}\,{\text{score}}\,\left( {28\,{\text{day}}} \right) = \left( {10^{4} } \right)\,*\,\exp \left( {\left( {0.048728*A} \right) + \left( {0.2005776*S} \right) + \left( { - 0.3591817*T} \right) + \left( {0.0098462*{\text{HR}}} \right) + \left( { - 0.0125259*H} \right)} \right)$$

While haematocrit and high heart rate did not offer independent predictive power in the 28-day mortality model, they were useful in explaining variability when retained in the model.

#### Comparison of the prognostic models with preexisting scores

Comparison of the prognostic models with SOFA and APACHE II was performed graphically by drawing the superimposed ROC curves and testing the underlying AuROC obtained, taking into account that the data are correlated, using a nonparametric approach as suggested by DeLong et al. [23].

For all statistical analyses, Stata version 10.0 was used (StataCorp, Texas, USA; http://www.stata.com).

## Results

### Baseline and outcome data

The derivation cohort included 462 patients with FP recruited in the UK. Their median (inter-quartile range, IQR) age was 69.4 (58.6–77.2) years. The geographic validation (non-UK) cohort included 515 FP patients recruited to the GenOSept study from the other European countries. Their median (IQR) age was 69.1 (58–77) years. The temporal validation cohort included 323 FP patients recruited in the UK between January 2011 and March 2015. Their median (IQR) age was 68.3 (57.6–77.2) years. For details of the recruiting centres, please see Additional file 1.

The baseline characteristics and the outcomes of the three cohorts are presented in Tables 2 and 3, respectively.

**Table 2:** Patients’ baseline characteristics for the derivation, geographic and temporal external validation sub-cohorts

Cohort	Derivation (UK until Jan 2011)		Geographic validation (non-UK)		Temporal validation (UK post-Jan 2011)	
Total number of patients	462		515		323	

Characteristics	Median or n	IQR or %	Median or n	IQR or %	Median or n	IQR or %
*Age*						
Available data	462	100%	515	100%	323	100%
18–34	11	2.4%	25	4.9%	11	3.4%
35–44	15	3.3%	18	3.5%	16	5%
45–54	54	11.7%	52	10.1%	38	11.8%
55–64	93	20.1%	98	19%	73	22.6%
65–74	113	24.5%	151	29.3%	88	27.2%
75–84	149	32.3%	149	28.9%	75	23.2%
85–95	27	5.8%	22	4.3%	22	6.8%
*Gender*						
Available data	462	100%	515	100%	323	100%
Male	236	51.1%	304	59%	171	52.9%
Female	226	48.9%	211	41%	152	47.1%
*Race*						
Available data	460	99.6%	510	99%	323	100%
Caucasian	454	98.7%	502	98.4%	315	97.5%
Asian	4	0.9%	7	1.4%	3	0.9%
African	1	0.2%	1	0.2%	3	0.9%
Mixed	1	0.2%	0	0%	2	0.6%
*Medical comorbidities*						
Available data	462	100%	515	100%	323	100%
Heart and vascular disease	187	40.6%	202	39.2%	117	36.2%
Respiratory disease	111	24.1%	133	25.8%	97	30%
Neurological disease	48	10.4%	57	11.1%	24	7.4%
Severe renal disease	39	8.6%	21	4.3%	16	5%
Gastrointestinal disease	98	21.3%	132	25.7%	76	23.5%
Malignancy	135	29.3%	160	31.1%	84	26%
Diabetes	61	13.2%	102	19.8%	44	13.6%
Previous serious infection^a^	8	1.7%	25	4.9%	5	1.6%
Other illness	130	28.2%	210	40.8%	83	25.7%
Severe exercise restriction	3	0.7%	6	1.2%	1	0.3%
Chronic dialysis	5	1.1%	8	1.6%	5	1.6%
Chronic steroids use^b^	2	0.4%	9	1.8%	5	1.6%
*Cause of FP*						
Available data	461	99.8%	511	99.2%	323	100%
Perforated diverticulum	137	29.7%	175	34.3%	89	27.6%
Anastomotic breakdown	115	25%	187	36.6%	61	18.9%
Malignancy	65	14.1%	64	12.5%	35	10.8%
Trauma	22	4.8%	45	8.8%	16	5%
Other	122	26.5%	40	7.8%	124	38.4%
*Time to surgery (days)*	1	1–3	1	1–3	1	1–3
*Acute physiology*						
Available data	461	99.8%	513	99.6%	321	99.4%
APACHE II score	15	12–20	17	13–22	16	12–21
SOFA score	7	5–9	7	5–11	6	5–8
Acute renal failure	129	32.7%	214	42.8%	70	21.8%
Renal replacement therapy	81	21%	105	21.3%	26	8.1%
Mechanical ventilation	346	75.1%	397	77.4%	228	71%

*APACHE* Acute Physiology and Chronic Health Evaluation, *SOFA* Sequential Organ Failure Assessment

^a^Serious infection was defined as a serious, prolonged or recurrent infection

^b^Chronic steroid use was defined as taking corticosteroids below the immunosuppression dose (>7 mg/kg/days hydrocortisone), which would exclude patient from inclusion in the study

**Table 3:** Outcomes for the derivation, geographic and temporal external sub-cohorts

Cohort	Derivation (UK until Jan 2011)		Geographic validation (non-UK)		Temporal validation (UK post-Jan 2011)	
Total number of patients	462		515		323	

**Characteristics**						
Length of stay (days)	Median	IQR	Median	IQR	Median	IQR
Available data	462	100%	515	100%	322	99.7%
ICU	7	4–14	14	7–29	6	3–11
Hospital	26	14–47	30	17–54	29	18–47

Mortality	*n*	%	*n*	%	*n*	%
Available data	462	100	515	100	321	99.4
6 month	124	26.8	185	35.9	64	19.9
ICU	73	15.8	131	25.4	24	7.5
Hospital	109	23.6	171	33.2	29	9.8
28 day	79	17.1	171	33.2	40	12.4

The age distribution was not significantly different across the cohorts, although the derivation cohort had a higher proportion of patients aged over 75. Males predominated in all cohorts. The racial distribution was more heterogeneous in the geographic validation cohort, while the derivation and the temporal validation cohorts were almost entirely Caucasian. Among the comorbidities diabetes, previous serious infections and other illnesses were more prevalent in the geographic validation cohort, compared to the other cohorts. The underlying causes for FP varied across cohorts, with anastomotic breakdown being particularly common in the geographic validation cohort. Baseline Sequential Organ Failure Assessment (SOFA) and Acute Physiology and Chronic Health Evaluation II (APACHE II) scores and prevalence of mechanical ventilation on day one were comparable across the cohorts. The occurrence of acute renal failure on day one was more frequent in the geographic validation cohort, with differences with the other cohorts (32.7, 42.8 and 23.3% for the derivation, geographic and temporal validation cohorts, respectively), accompanied by a difference in the utilisation of renal replacement therapy (21, 21.3 and 7.5% for the derivation, geographic and temporal validation cohorts, respectively) on day one. The geographic validation cohort was characterised by higher mortality rates (at all time points) and longer ICU stay, compared to the other two cohorts; this latter feature was also reflected, although to a lesser extent, in the length of hospital stay.

### Performance of the prognostic tools

**Fig. 1:**
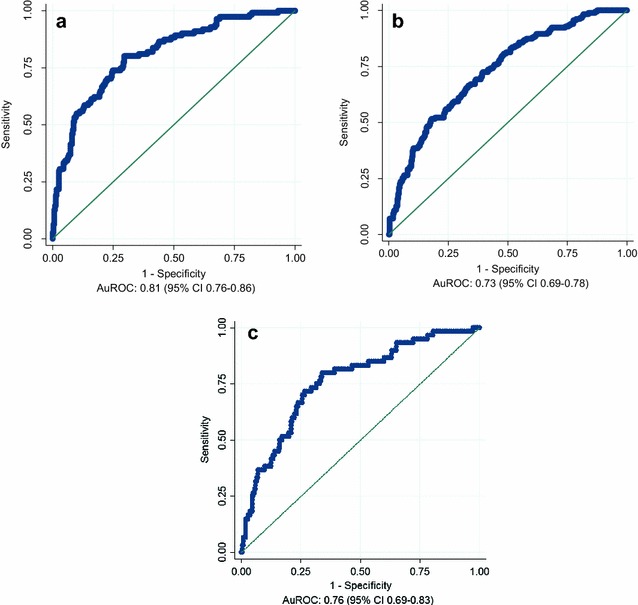
Receiver operating characteristics (ROC) curve obtained when applying the 6-month prognostic model to the derivation (**a**), geographic validation (**b**) and temporal validation sub-cohorts (**c**) respectively; *AuROC* area under the receiver operating characteristic curve, *CI* confidence interval

When evaluated using a receiver operating characteristics (ROC) curve, the discriminatory performance of the 6-month prognostic model in the UK derivation sub-cohort yielded an AuROC of 0.81 (95% CI 0.76–0.86) as indicated in Fig. 1a. At geographic validation in the non-UK sub-cohort, the 6-month prognostic model produced an AuROC of 0.73 (95% CI 0.69–0.78; Fig. 1b). At temporal validation, the 6-month model yielded an AuROC of 0.76 (95% CI 0.69–0.83; Fig. 1c).

**Fig. 2:**
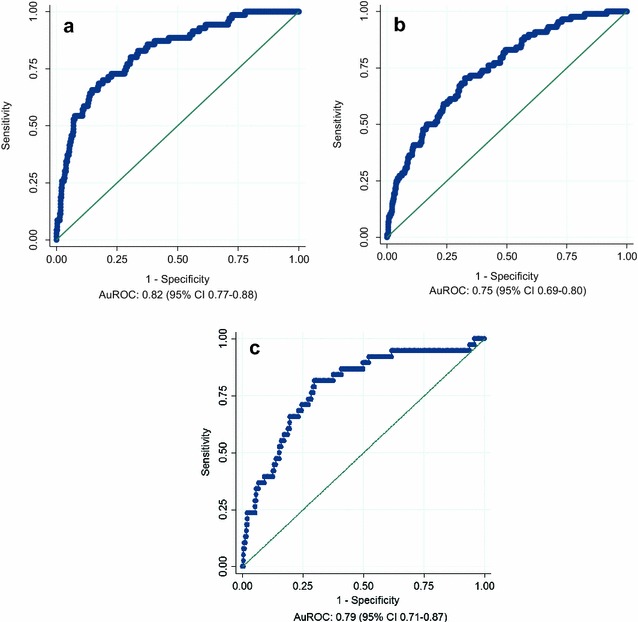
Receiver operating characteristics (ROC) curve obtained when applying the 28 day prognostic model to the derivation (**a**), geographic validation (**b**) and temporal validation sub-cohorts (**c**) respectively; *AuROC* area under the receiver operating characteristic curve, *CI* confidence interval

The 28-day prognostic tool also performed similarly, yielding an AuROC 0.82 (95% CI 0.77–0.88; Fig. 2a) for the derivation UK sub-cohort. At geographic validation in the non-UK sub-cohort, the 28-day prognostic model produced an AuROC of 0.75 (95% CI 0.69–0.80; Fig. 2b). In the temporal validation cohort, the 28-day model yielded an AuROC of 0.79 (95% CI 0.71–0.87; Fig. 2c).

The 6-month FP prognostic score produced numerical values which can be stratified within 5 intervals (0–2; above 2–4; above 4–6; above 6–12; above 12) corresponding to five levels of 6-month mortality risk. The 28-day mortality FP score produces values classified within 5 intervals, corresponding to different risk categories for the outcome (0–2; above 2–4; above 4–8; above 8–16; above 16). The observed mortality rates corresponding to each class of risk for the two scoring systems are presented in Table 4 for all three cohorts (Additional file 1: Figs. S1 and S2 display the corresponding histograms of mortality). A 6-month FP score above 12 is consistently associated with a greater than 50% mortality risk at 6 months across all cohorts. A 28-day FP score above 16 is associated with a greater than 40% mortality risk for the 28-day outcome for the derivation and geographic validation cohorts, but not for the temporal validation cohort, in which the highest observed mortality risk was around 22%.

**Table 4:** Observed 6-month and 28-day mortality rates for the derivation, geographic and temporal external validation sub-cohorts, stratified by FP score interval

Cohort	Derivation (UK until Jan 2011)	Geographic validation (non-UK)	Temporal validation (UK post-Jan 2011)
FP score	Deceased	Deceased	Deceased
6-month mortality			
0–2	3 (3.7%)	14 (13.7%)	5 (6.3%)
>2–4	11 (10.8%)	25 (22.5%)	7 (10.5%)
>4–6	14 (20%)	29 (36.3%)	12 (26.1%)
>6–12	29 (31.9%)	44 (40.7%)	15 (28.9%)
>12	67 (57.3%)	73 (64%)	22 (59.5%)
28-day mortality			
0–2	0 (0%)	10 (9.9%)	2 (2.7%)
>2–4	8 (8.3%)	12 (12%)	3 (5.4%)
>4–8	10 (9.5%)	17 (15.3%)	8 (11.1%)
>8–16	14 (16.5%)	27 (26.2%)	12 (22.2%)
>16	47 (45.6%)	42 (42%)	15 (22.4%)

### The discriminatory capabilities of the FP prognostic tools versus the SOFA and APACHE II scores in the FP cohorts

To assess how the FP models compare, as prognostic tools, to the routinely used SOFA and APACHE II scores, we calculated AuROCs for these scoring systems, to predict 6-month and 28-day mortality, in order to compare each tool across all cohorts and for both outcomes. For 6-month mortality, the SOFA score produced AuROCs of 0.73 (95% CI 0.68–0.78), 0.68 (95% CI 0.63–0.72) and 0.62 (95% CI 0.54–0.7) in the derivation, geographic and temporal external validation cohorts, respectively, while the APACHE II score yielded AuROCs of 0.74 (95% CI 0.7–0.79), 0.71 (95% CI 0.66–0.75) and 0.69 (95% CI 0.62–0.77) for those cohorts, respectively. For the 28-day mortality outcome, the SOFA score produced AuROCs of 0.76 (95% CI 0.7–0.82), 0.66 (95% CI 0.6–0.73) and 0.67 (95% CI 0.58–0.77) in the derivation, geographic and temporal external validation cohorts, respectively, while the same AuROCs for the APACHE II score were 0.71 (95% CI 0.64–0.77), 0.69 (95% CI 0.63–0.75) and 0.75 (95% CI 0.67–0.83), respectively.

The AuROCs obtained using the FP scores were consistently superior to those obtained with the SOFA score, with statistical significance across all cohorts (derivation, geographic and temporal external validation) and for both 6-month and 28-day mortality outcomes (Additional file 1: Figs. S3 and S4, respectively).

The AuROCs obtained using the FP scores were also superior to those derived using the APACHE II score for both outcomes, although statistical significance was not consistently achieved across all cohorts (Additional file 1: Figs. S5 and S6, for 6-month and 28-day mortality, respectively).

## Discussion

Faecal peritonitis continues to be associated with a high mortality. Approximately one out of five critically unwell patients with FP in Europe will die in the intensive care unit; this mortality rate increases to over 30% at 6 months.

As we previously reported, and perhaps unexpectedly, the presence of co-morbidities, the time from presumed onset of symptoms to surgery, the underlying cause of FP and the degree of organ support needed in critical care did not appear to influence survival significantly in these postoperative critically ill patients [24, 25]. We are not aware of any prognostic tool designed to assess the risk of long-term mortality specifically in the critically ill postsurgical FP patient. The risk prediction models described in our study aim to improve the SOFA score’s predictive power for mortality at 6 months and 28 days, by adding just a few key variables: age, lowest recorded temperature, highest recorded heart rate and haematocrit on admission to intensive care.

The 6-month mortality model demonstrates AuROCs of 0.81 (0.76–0.86), 0.73 (0.69–0.78) in the derivation and geographic validation cohorts, respectively, while the 28-day prognostic tool yielded AuROCs of 0.82 (0.77–0.88) and 0.75 (0.69–0.80) for the same cohorts. An area under the ROC curve over 0.8 is generally regarded as indicating a good discriminatory capacity [26]. In the temporal validation cohort, the 6-month and 28-day mortality models yielded AuROC of 0.76 (95% CI 0.69–0.83) and 0.79 (0.71–0.87), respectively. The models, therefore, retained reasonable discriminatory capability, and systematically outperformed the other scoring systems tested (SOFA and APACHE II), in these cohorts.

This FP prognostic tool may, therefore, be useful to complement the currently used risk scores and bedside clinical assessment, enhancing the critical care clinician’s capacity to predict long-term outcome, thereby supporting the clinical decision making process in the postoperative phase.

The prognostic models presented here have some strengths, particularly as they have been derived and internally validated using large, homogeneous and recently gathered cohorts of FP patients (hence reflecting current practices and therapies).

Biondo and colleagues have recently evaluated the performance of the MPI as a predictor of immediate postoperative mortality, demonstrating an AuROC of 0.72 (95% CI 0.65–0.79), while, for the more specific left colonic Peritonitis Severity Score (PSS), the AuROC was 0.79 (95% CI 0.72–0.85) for this outcome [27].

We have previously reported that factors such as age, acute renal dysfunction, hypothermia, lower haematocrit and thrombocytopaenia are associated with an increased risk of death from FP [24, 25], and a number of other studies have evaluated the prognostic relevance of the individual components of our proposed prognostic models.

### SOFA

The SOFA score was developed in a mixed (medical and surgical) ICU population [28] and has been subsequently externally validated in various populations [1], such as cardiac surgical patients [29] and critically ill burn patients [30].

While the SOFA score was originally developed for the purpose of describing the evolution of organ dysfunction, rather than for prognostic purposes, we previously found that both admission SOFA and trends in the global SOFA scores were closely associated with mortality [25]. Many studies have reported the use of the SOFA score both in isolation [31–35] and in combination with other variables [36, 37], for the purpose of outcome prediction. In our study, neither the SOFA nor the APACHE II scores, when used in isolation, performed as well as the tools developed here. Furthermore, day one SOFA performed particularly poorly in the temporal validation group, while the APACHE II risk model (which was developed for the purpose of outcome prediction) performed more consistently across the three cohorts, both for the 6-month and the 28-day outcome. This finding suggests that the value of SOFA lies primarily in describing temporal changes in organ function. Nevertheless, a single SOFA score can be successfully integrated with other parameters, to provide a prognostic tool with improved accuracy [36, 37], as we have done for day one SOFA in these analyses. While the confidence intervals for the AuROCs were relatively wide, when the FP models were compared to SOFA, statistically significant differences were found across all cohorts. This was not always the case for comparisons with APACHE II, further highlighting the superior prognostic accuracy of this severity score compared to an isolated, day one SOFA score.

### Hypothermia

The adverse effect of hypothermia on the outcome of critically ill patients has been described by other authors, although data on the relevance of hypothermia to outcomes remain conflicting [38, 39]. Laupland and co-authors studied 10,962 medical, non-scheduled and scheduled surgical patients admitted to critical care with varying degrees of hypothermia and fever. Hypothermia was, after controlling for confounding factors, significantly and independently associated with mortality in medical patients [38]. Tiruvoipati et al. reported data from 175 elderly ICU patients, identifying lower temperatures and the Simplified Acute Physiology Score II (SAPS II) during the first day of ICU admission as being independently associated with higher hospital mortality [39, 40]. An association between severe hypothermia and the risk of ICU acquired infections has also been reported among medical patients [41].

### Highest recorded heart rate

An increased heart rate is a physiological response to infection and sepsis, and part of the systemic inflammatory response syndrome (SIRS). Sprung and colleagues found that the presence of SIRS predicts infection, severity of illness, organ failure and outcome, with the two most common SIRS criteria met during ICU stay being respiratory rate (82%) and heart rate (80%) [42]. Morelli and co-workers randomised a total of 154 septic shock patients to receive a continuous infusion of esmolol (targeting a heart rate of 80–94 bpm) or standard treatment in an open label trial. The patients in the esmolol arm achieved lower heart rates, without an increase of adverse events. Interestingly, an improvement in survival and other secondary outcomes was also reported [43]. Others have found that a high daily mean heart rate was a significant predictor of ICU mortality [44].

### Haematocrit

Anaemia in surgical patients undergoing both cardiac and non-cardiac procedures has previously been reported to be associated with worse outcomes [45–49]. Beattie and co-workers performed a retrospective observational study of 7759 non-cardiac surgical patients to establish the relationship between preoperative anaemia and postoperative mortality and found that preoperative anaemia was common and strongly linked with postoperative mortality, even after adjustment for major confounders [49].

All of the patients with FP included in the analyses reported here underwent laparotomy (the diagnosis of FP was based on the intra-operative finding of faecal soiling of the peritoneal cavity). In addition, a significant proportion of patients (40%) were documented to have cardiovascular co-morbidity, a group in which anaemia has been shown to be associated with worse survival and major adverse cardiovascular events. Although anaemia may be associated with a poor outcome, data on the effects of blood transfusion are conflicting, with most reports not demonstrating benefit from transfusion aimed at achieving a higher haemoglobin threshold [50, 51].

### Limitations

One limitation of the current study is that we were unable to test the performance of other scoring systems such as the colorectal POSSUM, the MPI, PIA II or the PSS in our dataset, as these systems all require some intra-operative or preoperative findings, which were not available to us. On the other hand, the fact that our scores do not require any intra-operative findings could be viewed as an advantage.

A further limitation is the lack of comparison with alternative and more recent versions of severity scores, such as the Simplified Acute Physiology Score (SAPS) 3, the APACHE III or IV or the Mortality Prediction Model (MPM) III. We consider this unlikely to have a significant impact on the validity of our results, as multiple studies have shown that the performance of such tools, even in their more recent versions, is not significantly improved [52]. A pragmatic decision was made to rely on the APACHE II (rather than more recent versions of APACHE) in view of its practicality, the fact that it is the only available non-proprietary version in widespread clinical use [1, 2, 4] and the comparator of choice in multiple other recently published studies [53, 54].

The SOFA score may be a less than ideal comparator, as the SOFA was not originally developed for prognostication. Multiple previous studies have, however, reported using the SOFA score, both in isolation [31–35] and in combination with other parameters [36, 37], for outcome prediction.

Another limitation is that our study was not designed to evaluate the influence on outcome of the timing and adequacy of source control or antibiotic treatment. All patients included in the study reported here received source control via surgical laparotomy prior to recruitment and the overwhelming majority of the patients (91.8%) received antimicrobial therapy deemed to be adequate [24].

Although the homogeneity of the patient population within our cohorts represents a methodological strength of the study, it may also be considered a potential weakness, as some *real-world* critically ill patients with FP would have not been included in our analyses.

Mortality differed markedly between the cohorts, even though they were recruited using the same inclusion and exclusion criteria. Whilst it is impossible to identify with certainty which factors explain these differences, multiple potential reasons can be postulated. Firstly, the variation in mortality rates strongly correlates with the occurrence of acute renal failure on day one. Acute renal dysfunction and deteriorating renal function have both been consistently associated with poor outcome in this specific subset of patients [24, 25]. The effects of random variability and the fact that in the UK the centres recruiting to GenOSept and those recruiting to GAinS were not always the same may have also contributed. Finally, improvements in the management of sepsis over the years may have influenced the incidence of renal failure and outcomes.

## Conclusions

The present study describes the development of two prognostic models for the risk of 6-month and 28-day mortality in critically ill septic patients with FP, following laparotomy for source control. The tools incorporate five of the major independent risk factors identified in previous studies (SOFA score, age, heart rate, temperature and haematocrit) and combine them to produce a numerical value associated with mortality risk over 6 months or 28 days. Although, in the setting of postoperative FP patients admitted to critical care, the tools appeared to be superior to other existing scoring systems, such as SOFA and APACHE II, these findings should not be considered definitive. External validation in larger cohorts, such as the NELA (National Emergency Laparotomy Audit) or other databases [55], of their predictive capability is needed before introduction of the scores into clinical practice to inform decision making and the design of clinical trials.

## Additional file

**Additional file 1.** Derivation and validation of a prognostic model for postoperative risk stratification of critically ill patients with faecal peritonitis.

## Abbreviations

APACHE: Acute Physiology and Chronic Health Evaluation
ARF: acute renal failure
bpm: beats per minute
CI: confidence interval
CPAP: continuous positive airways pressure
CVS: cardiovascular
CXR: chest radiography
eCRF: electronic case report form
FP: faecal peritonitis
GCS: Glasgow Coma Scale
HR: hazard ratio
ICU: Intensive Care Unit
IQR: interquartile range
MAP: mean arterial pressure
MOSF: multiple organ system failure
N: number of non-missing observations
paO2: arterial partial pressure of oxygen
paCO2: arterial partial pressure of carbon dioxide
P:F: ratio of partial pressure arterial oxygen and fraction of inspired oxygen
RRT: renal replacement therapy
SBP: systolic blood pressure
SOFA: Sequential Organ Failure Assessment
WCC: white cell count
